# REBT with Members of the Church of Jesus Christ of Latter-day Saints

**DOI:** 10.1007/s10942-023-00524-z

**Published:** 2023-09-27

**Authors:** Stevan Lars Nielsen, Brodrick T. Brown, Dane D. B. Abegg, Dianne L. Nielsen

**Affiliations:** 1 Counseling and Psychological Services, Brigham Young University, 1500 WSC, BYU, Provo, UT 84602 USA; 2 Counseling Psychology and Special Education, Brigham Young University, Provo, UT 84602 USA; 3 Kansas City VA Medical Center, Kansas City, MO 64128 USA; 4 Private Practice, Orem, UT 84097 USA

**Keywords:** Religion, Rational emotive behavior therapy, Psychotherapy effectiveness

## Abstract

Rational Emotive Behavior Therapy’s (REBT’s) ABC model proposes that it is B, Beliefs about A, Activating events, not A, Activating events themselves, that create and control C, emotional Consequences. Codified beliefs such as scriptures and creeds are prominent in most religions. Integrating codified religious beliefs with REBT to help D, Dispute irrational beliefs has been studied in REBT for more than 50 years. Broad knowledge of religious cultures, scriptures, creeds, and wisdom literature is likely to help REBTers and other cognitive behavior therapists (CBTers) more effectively treat religious clients. We give a brief overview of the history, culture, doctrine, and scriptures of the Church of Jesus Christ of Latter-day Saints, then give examples of REBT Disputations excerpted from religion integrative sessions with practicing Latter-day Saint clients. We present practice-based evidence for the effectiveness of this approach and offer suggestions for future study and research in integrating religion with REBT and CBT.

## Introduction

Even as Albert Ellis attacked religion (Ellis, 1971, 1980), other rational emotive behavior therapists (REBTers) showed that religion can counter irrational beliefs (iBs; Hauck, 1972; Hauck & Grau, 1968; Robb, 1988; Young, 1984). Clinical trials (e.g., Johnson et al., 1994; Johnson & Ridley, 1992; Propst, 1980a, 1980b; Propst et al., 1992) and meta-analyses (Captari et al., 2018; Smith et al., 2007) show that religious and spiritual techniques (RSTs), including “Biblical disputation” (Smith et al., p. 647), are as effective as secular interventions at reducing religious clients’ symptoms and more effective at improving religious clients’ sense of wellbeing. Ellis remained an atheist, but softened his approach toward religion (Ellis, 2000), co-authoring a book about REBT for religious clients (Nielsen et al., 2001). REBTers have attempted to make REBT sensitive to religious diversity: using the Bible with “Bible Belt” Christians (Young, 1984); searching the Qur’an to dispute a Muslim client’s iBs (Nielsen, 2004); tailoring REBT for Christian, Jewish, and Muslim couples (Johnson, 2013); and using Jewish scripture and wisdom literature to help Jewish clients control and overcome anger (Schiffman, 2016). We describe the Church of Jesus Christ of Latter-day Saints and give examples of RSTs used with observant Latter-day Saint clients.

## The Church of Jesus Christ of Latter-day ﻿Saints

The Church of Jesus Christ of Latter-day Saints,^1^ headquartered in Salt Lake City, Utah, had 16.8 million members in 161 countries in 2022. For the 12 years before the COVID-19 pandemic the Church grew by approximately 250,000 converts annually (Church of Jesus Christ, 2022a). The Church’s growth makes it likely that therapists will have Latter-day Saints among their clients. Members who seek therapy are encouraged to look for credentialed professionals who are respectful of religion (Church of Jesus Christ, 2022g). Therapists known to be knowledgeable and respectful of the Church are more likely to have Latter-day Saints seek their help.

### History

Latter-day Saints believe Jesus Christ’s New Testament Church was lost to apostasy after the early Apostles died and that restoration of the original Church of Jesus Christ began in 1820 with the appearance of God the Father and Jesus Christ to 14-year-old Joseph Smith. Several ancient prophets subsequently returned as resurrected angels to pass on their specific authority or *keys* as part of this restoration. Members believe the Restoration is an ongoing, revelatory process (*Pearl of Great Price*—*PGP*, 1842/2013, Article of Faith 9).^2^ The Church was formally organized in 1830. *Latter-day* refers to the current era, believed to precede Christ’s second coming—Church leaders make no specific predictions about when this will occur. *Saints* refers to ordinary Church members, not venerated or canonized individuals (*Doctrine and Covenants*—*D&C*, 1838/2013, 115).

#### Conflict

Unorthodox theology, early, rapid growth with centralized gathering of converts, and internal dissent led to sometimes violent conflict that forced the Church from New York and Pennsylvania to Ohio, Missouri, Illinois, and then to what is now Utah. Joseph Smith was shot to death by a mob in 1844 while under a guarantee of protective custody in an Illinois jail. Facing further violence, the Church’s governing Quorum of Twelve Apostles and its second Prophet, Brigham Young, moved to the Great Basin, then part of northern Mexico. Salt Lake City was founded in 1847. In 1850, after the Mexican–American war, the US annexed the area and created Utah as a slave-holding territory, appointing Brigham Young its first territorial governor.^3^

In response to tensions associated with the Church's practice of polygamy, the US government appointed a new governor and in 1857 sent 2,500 federal troops to enforce federal control. Utah became a hub in the western US migration after the US Civil War. Anti-polygamy laws passed in 1887 imprisoned polygamous men, disenfranchised polygamous women, and confiscated Church property (Arrington & Button, 1992). In 1890, Wilford Woodruff, the Church’s fourth Prophet, ended polygamous marriage with a proclamation that is canonized (*D&C*, 1890/2013, Proclamation 1). Utah became the 45th US state in 1896. The Utah State Constitution forbids polygamy: “Perfect toleration of religious sentiment is guaranteed. No inhabitant of this State shall ever be molested in person or property on account of his or her mode of religious worship; but polygamous or plural marriages are forever prohibited” (Utah const. art. III, § 1).^4^

This history of conflict is part of the Church’s official history, taught to most members, including new converts. It can contribute to an us-versus-them mindset among members. A sense of persecution, combined with word-of-mouth reports of therapist dismissiveness toward religion (cf. Trusty et al., 2022; Yamada et al., 2020) can interfere with Latter-day Saints developing a therapeutic alliance with non-member therapists.

#### Integration and Acceptance

By 1965 there were more Latter-day Saints outside than in Utah and by 1990 more outside than in the US. Members are instructed to share their beliefs with others. For example, Marriott International, one of the world’s largest hoteliers (CEOWorld Magazine, 2022), founded by Latter-day Saints, J. Willard and Alice Marriott, allows placement of copies of the *Book of Mormon* with copies of Gideon Society Bibles in many of its hotel rooms. The Church is familiar enough to be a target for popular satire: The musical, “The Book of Mormon” (Parker et al., 2011), has satirized the Church on Broadway and London’s West End for 12 years (Peikert, 2019). The Church has responded by purchasing advertisements for the Church and for the *Book of Mormon* in *Playbill* programs for the play, including one add that read, “You’ve seen the play, now read the book!” (Walker, 2012).

The Church encourages members to obtain higher education and contribute to society. Its colleges and universities have a residential enrollment of 56,000, plus growing numbers of students; currently approximately 45,000 students are in accredited online programs available in the USA and internationally (Church of Jesus Christ, 2022f). Brigham Young University (BYU), in Provo, is the third largest private US university, and currently ranks sixth among US universities for sending undergraduates on to earn doctorates; it is third for sending undergraduates on for doctoral degrees in psychology (Kang, 2022). The Church endorses scientific medicine, including vaccination (Church of Jesus Christ, 2022g). Before joining full-time Church leadership roles, the Church’s governing First Presidency, the Prophet and his two counselors, were a heart surgeon, a state Supreme Court Justice, and a Stanford  business professor.

The Church gained political prominence from 1953 to 1961 when Ezra Taft Benson served both as one of the Church’s governing Apostles and as a Cabinet member for US President Dwight Eisenhower. Latter-day Saint and Republican Mitt Romney lost the 2012 US presidential election to Barack Obama. Now a Republican senator representing Utah, Romney has remained politically prominent because of his ongoing harsh criticism of Republican Donald Trump. He twice broke with other Republican Senators to support impeachment of then President Trump and recently characterized Trump as “hanging over [the Republican Party] like a gargoyle” (Nelson, 2022). On the opposite side of the US political spectrum, Democrat and Latter-day Saint Harry Reid represented Nevada in the US Congress and served as US Senate Majority Leader from 2007 to 2015. Church Apostle, M. Russell Ballard, who is third in the Latter-day Saint leadership hierarchy, joined US President Joseph Biden and former President Barack Obama in giving a eulogy at Reid’s Las Vegas funeral (Walch, 2022).

### Latter-Day Saint Scripture

The Latter-day Saint canon includes the *King James Version* of the Bible (*KJV*), the *Book of Mormon: Another Testament of Jesus Christ* (*BofM*), the *Doctrine and Covenants* (*D&C*), and the *Pearl of Great Price* (*PGP*). Latter-day Saint Apostles are considered prophets who are expected to lead the Church through a process of ongoing revelation (*PGP*, 1842/2013, Article of Faith 9). Members are instructed to seek revelation to guide their own lives. Scriptures, Church manuals, and governing policy statements are available at www.churchofjesuschrist.org.

The Bible is accepted as God’s word insofar as its translation and provenance are correct (*PGP*, 1842/2013, Article of Faith 8). Non-English speakers use agreed upon, scholarly Bible translations.

Published in 1829, the *BofM* is accepted as God’s word (*PGP*, 1842/2013, Article of Faith 8). Joseph Smith said that in 1827 an angel named Moroni gave him ancient records on golden plates, sections of which Smith translated through divine inspiration. The *BofM* is approximately one-third the length of the *KJV*. It is named for Mormon, a prophet in the ancient Americas who excerpted and summarized writings of ancient prophets in the Americas. The *BofM* tells of Christ’s visit to the Americas after his resurrection. Mormon’s son Moroni added to and stored the plates, returning as a resurrected angel to entrust the plates to Smith until he finished translating the parts of the plates assigned to him (*PGP*, 1834/2013, Joseph Smith—History). Statues atop many Latter-day Saint temples depict Moroni as an angelic herald of Christ’s second coming.

At 60% the length of the *KJV New Testament* (*NT*), the *D&C* contains 136 of Joseph Smith’s revelations and four revelations given to other Church Prophets in 1847, 1890, 1918, and 1978. Members believe new revelations will be added to the *D&C* as Christ deems necessary.

The *PGP* is slightly longer than Luke in the *KJV NT.* The *PGP* contains Joseph Smith’s account of his earliest revelations, 13 Articles of Faith, and revelations shown to Joseph Smith by God that were originally given to Enoch, Abraham, and Moses, then  lost from Genesis.

### Latter-Day Saint Theology

#### The Plan of Happiness

**Fig. 1:**
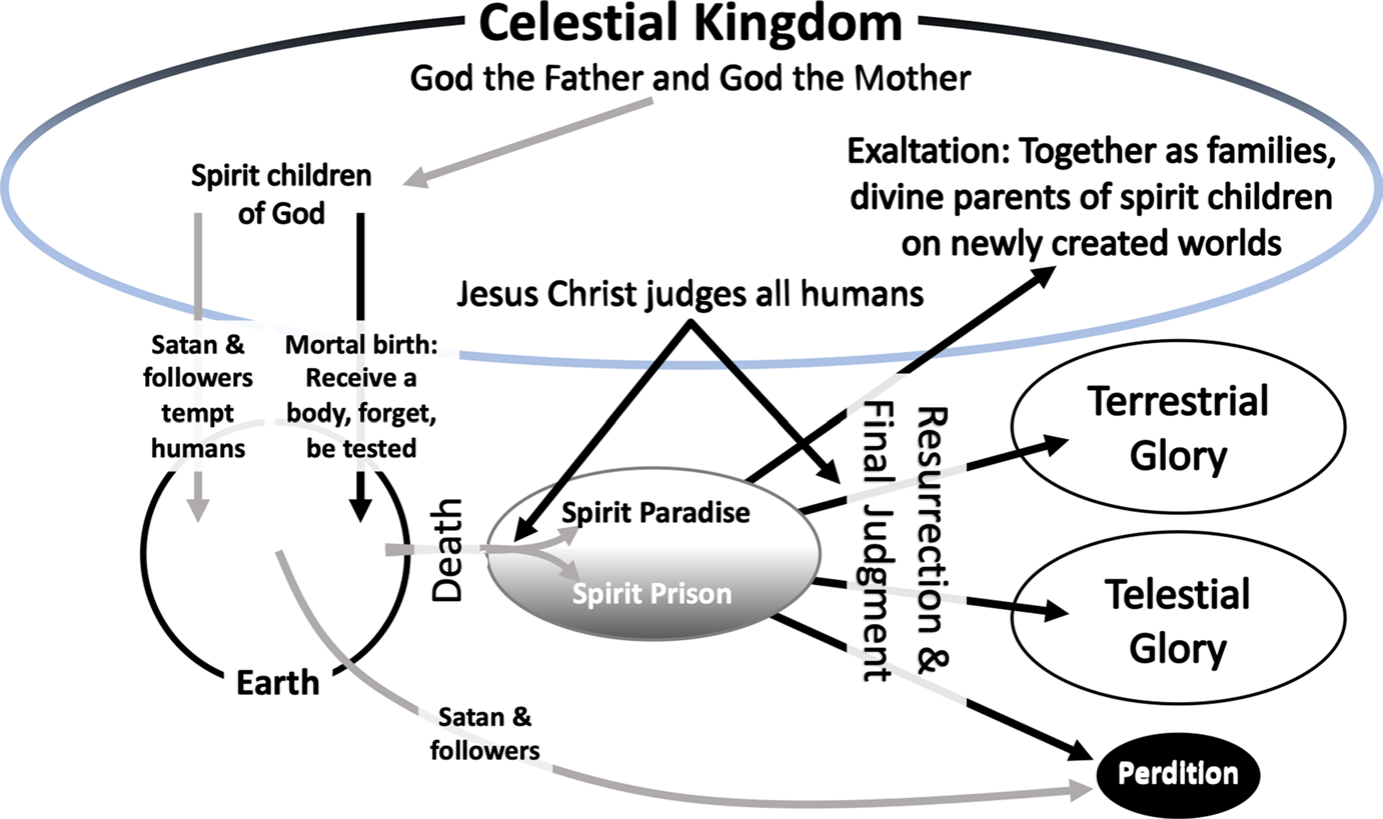
The Plan of Happiness: Latter-day Saint Model of Creation and God’s Children, Including Angels, Humans, and Devils, With Their Developmental Sequence

The *PGP* records that God told Moses, “This is my work and my glory: to bring to pass the immortality and eternal life of man” (*PGP*, 1830/2013, Moses 1:39). Lorenzo Snow, the fifth Prophet, wrote, “As man now is, God once was: As God now is, man may be” (Church of Jesus Christ, 2012, p. 84). A prophet named Lehi in the *BofM* told his son, Jacob, “Adam fell that men might be; and men are that they might have joy” (*BofM*, 1830/2013, 2 Nephi 2:25). God's Plan of Happiness is to give humans who are willing to accept the associated responsibilities of godhood an eternally joyful existence like God’s own existence (Church of Jesus Christ, 2022d; see Fig. 1).

#### Deity

God the Father, God the Mother, Jesus Christ, and the Holy Ghost are separate, deific beings who are united in their goals. They have emotions. God the Father, God the Mother, and Christ have physical bodies; the Holy Ghost is a spirit. Spirits look like humans, but are incorporeal (*D&C* 1831/2013, 129). Christ, humans, Satan, and his followers are spirit children of God the Father and a Mother in Heaven who is also a God. Mother in Heaven is not otherwise explained in Church theology. Members are instructed not to pray to her. Christ, humans, angels, and Satan and his followers were spirits before the world was created. Satan and his followers rebelled, were cast out of God’s presence, and lost forever the chance to have physical bodies. (Church of Jesus Christ, 2022i; 2022j; 2022l; see Fig. 1). Angels who visit humans may be pre-mortal spirits or resurrected beings like Moroni.

#### Jesus Christ

Jesus Christ is God the Father’s firstborn spirit child. Christ created the earth and was Jehovah, the God of the Old Testament while still a premortal spirit (*BofM*, 1830/2013, Mosiah 3:8). As the physical son of Mary and the divine son of God the Father, Christ grew into and obtained divine authority and power (D&C, 1831/2013, 93:11–13). Latter-day Saint theology states that physical and spiritual salvation are gifts of grace from Jesus Christ (*BofM*, 1830/2013, 2 Nephi 25:24).

#### Physical Salvation

Christ willingly allowed his own physical suffering and death. He then used his divine power to reunite his spirit with a perfect body in the first resurrection. All humans will be resurrected as a free gift of grace from Christ (Church of Jesus Christ, 2022b).

#### Spiritual Salvation

Christ endured all possible mortal suffering, including all possible physical pain from illness and injury. He experienced all possible emotional pain that can arise as part of the human condition, including discouragement, fear, loneliness, and so on. He experienced all emotional pain that can arise from others’ sins, including from betrayal, deceit, mockery, and so on. Because Christ was perfectly empathic, he experienced all emotional pain that can arise from guilt. Christ experienced this suffering but neither sinned nor used his divine power to ease his own suffering. Christ’s innocent suffering gives him complete empathy for humans, allows him to forgive sin, and empowers him to reunite humans with God the Father. The spiritual salvation Christ offers requires that humans accept him as God and Savior and that they strive to repent, strive to serve others, and strive to obey Christ’s commandments (Church of Jesus Christ, 2022b; 2022i).

#### The Holy Ghost

The Holy Ghost is a spirit with divine power, especially power to give humans spiritual experiences. He bears witness of the Father, Jesus Christ, the Plan of Happiness, and other truths. His identity and destiny are not otherwise known (Church of Jesus Christ, 2022h). Church membership requires baptism and receiving the gift of the Holy Ghost when confirmed a member of the Church. This gift is a special right to receive revelatory communication through the Holy Ghost (*PGP*, 1842/2013, Article of Faith 4). Members aspire to spiritual gifts that come through the Holy Ghost, including dreams, visions, healing, tongues, and especially revelation pertinent to one’s responsibilities and personal life (*PGP*, 1842/2013, Article of Faith 7). Members pray for spiritual help in solving problems. We find that our Latter-day Saint clients are prone to anxiety arising from a demand for spiritual confirmation of their decisions, though scripture also tells them to act as “agents unto themselves” (*D&C*, 1831/2013, 58:28).

#### Satan and his Followers

Satan and his followers, all spirit siblings of humans, are the only demons in Latter-day Saint theology. One third of pre-mortal spirits, they were cast out of God’s presence for stubbornly rejecting agency in the Plan of Happiness. They are motivated by a kind of spiteful, misery-loves-company goal to tempt humans away from God (Church of Jesus Christ, 2022d).

#### Humans

Humans are eternal; they had an existence before they were spirit children of God the Father and God the Mother, though this is not further explained in Church theology.

#### Pre-Mortal Existence

All humans were present when the heavens and the earth were created. They agreed to and supported the Plan of Happiness. Humans have inklings of pre-mortal experiences, but to preserve moral agency they do not have clear memories of these experiences (Church of Jesus Christ, 2022l; see Fig. 1).

#### Mortality

God the Father and God the Mother have perfect physical bodies. Mortality embodies spirits, placing humans in the challenging physical world where they have freedom and moral agency to learn and act for themselves. The eternal spirit that enlivens the physical body learns from the physical experience of mortality. Humans are foreordained but not predestined to widely diverse mortal roles and settings. They will be judged for their responses to mortality. Especially important in judgment is how humans treat each other and how they respond to what they learn about Christ through the Holy Ghost’s revelations. Mortal choices provide the strongest evidence of a person’s desires and faith (Church of Jesus Christ, 2022d, 2022f; see Fig. 1).

*The Family: A Proclamation to the World*, clarifies Latter-day-Saint verities about mortality (Church of Jesus Christ, 1995): Gender is a pre-mortal trait. Sexual intimacy is acceptable only between a married man and woman. Family life can give humans the fullest possible joy; it is a simulacrum of God’s eternal life. Faithful marriages sealed by proper Church authority will last forever. Parents are equal partners; mothers are usually the primary nurturers of children; fathers are usually the primary providers for families. Parents and societies have a moral obligation to teach children right and wrong. Individuals and societies will suffer severe practical and spiritual consequences if they violate these principles. These negative consequences are usually natural outgrowths of violating eternal principles or laws.

#### Human Nature

Human nature arises through the interaction of an imperfect spirit with an imperfect body (*D&C*, 1833/2013, 88:15). Humans are innocent but fallible at birth and sin in response to appetites, passions, and Satanic temptations. Premortal and mortal traits, experience, and decisions create fallible human individuality (*BofM*, 1830/2013, Mosiah 3:19; *D&C*, 1833/2013, 93:38). Albert Ellis wrote, “People are born crazy—all of them!” (Dobbins, 2005). Similarly, a *BofM* prophet named Jacob said that if humans want to get into heaven they must “consider themselves fools before God” (*BofM*, 1830/2013, 2 Nephi 9:42).

#### Sin

For one to sin one must know right from wrong, though innocent misbehavior can still create serious consequences. Commandments protect from both the practical and spiritual harms of sin. The main spiritual harm of sin is separation from God (Church of Jesus Christ, 2022b). Humans are not evil because of Adam and Eve, but will inevitably sin. Children are innocent until accountable at around age eight, when they are to be baptized and confirmed. Young children and older children, teens, or adults with significant intellectual or developmental disabilities are saved and will be in what is called Celestial Glory or the Celestial Kingdom (*BofM*, 1830/2013, Moroni 8:10–21; *D&C*, 1831/2013, 68:25–27; see Fig. 1). Private repentance is sufficient for most sin, but restriction or loss of Church membership may follow sins that have significant negative effects for others or for the Church (Church of Jesus Christ, 2022g).

#### Word of Wisdom

The Word of Wisdom is a recommendation to strive for physical health as well as a commandment that declares use of alcohol, tobacco, coffee and tea—not caffeine, per se—sinful (*D&C*, 1833/2013, Sect. 89). Recreational drug use is also prohibited, though oral marijuana may be used where it is legal if it is prescribed by a credentialed physician (Church of Jesus Christ, 2022g).

#### Sexuality

Sexual desire and behavior are for enhancing the emotional intimacy of heterosexual marriage as well as for procreation. Marriage is a sacred blessing that can become eternal when solemnized in a Latter-day Saint temple through the proper priesthood authority. Sexuality in marriage, including birth control, is a private matter between a wife and husband (Renlund & Renland, 2020). Pornography and masturbation are considered sinful, but generally not matters for formal discipline. Sexuality outside marriage is considered serious sin. Divorce is not sinful, per se, but ending an eternal Temple marriage may restrict access to a future Temple marriage, especially if infidelity was a cause of the divorce (see Temple, below; Church of Jesus Christ, 2022g).

#### Abortion

The *BofM* suggests that ensoulment occurs first at birth (*BofM*, 1830/2013, 3 Nephi 1:13). Abortion for reasons of convenience is considered a very serious sin, though not equal to murder. If the decision is confirmed through careful, spiritually informed deliberation, abortion is allowed in cases of rape, incest, to protect the mother’s life, and in cases of serious fetal malformation (Church of Jesus Christ, 2022g).

#### Sexual Minorities

Homosexual behaviors, but not homosexual urges are considered sinful. Sexual minority labels and alternative gender pronouns are discouraged. Gender transitioning and reassignment may lead to membership limitations unless approved by the Church’s First Presidency (Church of Jesus Christ, 2022g). The Church opposed same-sex marriage and from 2015 to 2019 restricted the children of same-sex parents from being baptized until they reached age 18. The Church now officially supports sexual minority legal rights, but teaches that same-sex marriages and LGBTQ+ behavior will restrict eternal family blessings (Kemsley & Stack, 2022).

### Judgment

Jesus Christ will judge all humans by balancing justice, mercy, and the blessings humans demonstrate by their choices that they were willing to receive (*BofM*, 1830/2013, 2 Nephi 9:41). Judgment occurs first in a temporary post-mortal spirit realm and is finalized at the time of each person’s resurrection (see Fig. 1).

#### Post-mortal Spirit Realm

Physical death separates one’s spirit from the body. Spirits then enter a realm divided between a Spirit Paradise and Spirit Prison with some resemblance to popular depictions of heaven and hell. The Spirit Prison is punishing because guilt is inescapable without sincere repentence. Repentance brings relief from guilty suffering (*BofM*, 1830/2013, Mosiah 2: 36–41). Many humans have never and will never learn of Jesus Christ or the Plan of Happiness during their mortal lives. Between the time of his own death and resurrection Christ organized missionary work in the post-mortal realm (*D&C*, 1918/2013, 138:18–30). There will be multiple occasions during mortality and in the post-mortal realm for all humans to learn about Christ and the Plan of Happiness, with opportunities to exercise faith in Christ, repent, obey Christ’s commandments, and receive saving ordinances (Church of Jesus Christ, 2022b; 2022i). Human spirits leave the post-mortal spirit realm at the time of their resurrection, bringing them to the eternal blessings they were willing to receive.

#### Resurrection

Separation of the spirit from the body is burdensome for the dead (*D&C*, 1831/2013, 45:17). Christ was the first human resurrected, followed by other righteous individuals. The time of one’s resurrection depends on one’s faithfulness and one’s assignments after death. For example, Moroni was a resurrected angel when he delivered the *BofM* plates to Joseph Smith, while others may have an assignment to serve as missionaries in the spirit realm between death and their own resurrection. Sin and late repentance delay resurrection (*D&C*, 1832/2013, 76). Humans will be in one of four resurrected states. Except in very rare cases, punishments for sin will not be unending (see Fig. 1).

#### Perdition

A very few humans will be in Perdition. These appear to be those who received spiritually confirmed knowledge of Jesus Christ, then harmed others by obdurately fighting Christ and working to undermine the Plan of Happiness. They will be resurrected last. Latter-day Saints with clear, certain, spiritual knowledge of Christ who stubbornly rebel and harm others would be more likely to be in Perdition. This self-chosen, emotionally punishing existence is not otherwise described in scripture. Satan and his followers will be in Perdition as disembodied spirits (*D&C,* 1832/2013, 76:25–48).

#### Telestial Glory

Those with serious mortal sins who do not repent in the post-mortal realm are next to last to be resurrected. Their guilty anguish is relieved when they are resurrected and they will enter a peaceful, indescribably beautiful, heaven-like realm called the Telestial Glory (1 Corinthians 15:41, *KJV*). This existence is glorious, but those here will not be with God (*D&C*, 1832/2013, 76: 81–91& 98–113).

#### Terrestrial Glory

Those who lived honorably, who repented of their sins, but did not accept Christ’s true Church with its saving ordinances will be resurrected with Terrestrial Glory. Christ will visit them, making this more glorious than the Telestial Glory. They will not be with their Father and Mother in Heaven and will not be eternally united in marriage with eternal families (*D&C*, 1832/2013, 76: 71–80).

#### Celestial Kingdom

Those who were valiant for Christ, who completed necessary saving ordinances, who demonstrated they were willing to accept divine responsibilities will be *exalted* in the Celestial Kingdom. They will be with Father and Mother in Heaven, Jesus Christ, the Holy Ghost, and will be sealed eternally with faithful family members. They will be creators of worlds and parents of spirit children, maturing to continue the Plan of Happiness cycle they began before their birth, emulating Father and Mother in Heaven as heavenly parents (D&C, 1832/2013, 76: 50–70, 92–95).

This Latter-day Saint eschatology is multidimensional, but our experience is that Latter-day Saint clients can adopt an absolutistic view of judgment and life after death. Many of our Latter-day Saint clients believe that anything other than Exaltation in the Celestial Kingdom would be an unacceptable, awful, hell-like result, though this is not doctrinally supported.

### Church Organization

Congregations are called Wards with as many as 500 members or Branches with as few as 5 members; 5 to 12 Wards or Branches make up Stakes or Districts. Branches and Districts are usually smaller and less stable than Wards and Stakes and are more common where the Church is newer and has fewer members. The name *Stake* is symbolic of the stakes used to stabilize Israel’s Tabernacle tent (Isaiah 33:20; 54:2, *KJV*). The Church is now the tent of Zion. Unpaid, lay men lead Branches, Districts, Wards, and Stakes as Bishops or Presidents. Ward or Branch Councils, including women as well as men, meet at least monthly to discuss and guide congregational activities.

The Church maintains central membership records with each member’s name, sex, date of birth, ordinance dates, parents, spouses, children, current address, and Ward or Branch assignment based on current address. Members and missionaries work to find and support members. There were 3,498 Stakes, 520 Districts, and 31,315 Wards and Branches in April, 2022 (Church of Jesus Christ, 2022a, 2022g).

#### Clergy

Men are encouraged to join the Melchizedek Priesthood (MP) when 18, boys are encouraged to join the Aaronic Priesthood (AP) when 12. MP offices are Apostle, Seventy, Patriarch, Bishop, and Elder. AP offices are Priest, Teacher, and Deacon. Men and boys are said to *hold* the Priesthood. MP holders preside over the Church. MP and AP members are assigned to *minister to* or serve members and non-members in their Wards or Branches. Each Ward and Branch has a MP and AP quorums, each with a president and counselors.

The Church’s only paid clergy are its General Authorities: the Prophet and two counselors in the First Presidency, the Quorum of Twelve Apostles, the First Quorum of the Seventy, and the Presiding Bishopric. The 15 Apostles in the First Presidency and Quorum of the Twelve serve for life; other General Authorities serve until age 70 (Church of Jesus Christ, 2022d). There are currently 123 General Authorities. The Church’s leadership hierarchy is First Presidency, then Quorum of Twelve Apostles, then Quorum of Seventy and Presiding Bishopric, then Stake or District President, then Bishop or Branch President.

The Relief Society (RS) was founded as a women’s service organization in 1842. Women are invited to join RS when 18. Each ward has a woman RS President with two women counselors. RS members receive ministering assignments. Girls are encouraged to join Young Women (YW) when 12. A President, two counselors, and advisors, all adult women, teach and supervise YW in wards. Like AP boys, YW girls join RS sisters in ministering assignments. Children from 18 months to 11 years attend Primary classes each Sunday; there are also weekday play or educational activities as often as twice a month. A woman President and two counselors lead Primary. Teaching is guided by a Sunday School President and two counselors who are men. The Sunday School organizes classes, reviews teaching, and provides in-service training (Church of Jesus Christ, 2022g).

Members view their local, lay leaders as temporary, rotating clergy. MP members also conduct priesthood ordinances for family members and, when needed, for neighbors to whom they minister. The preferred pattern is for fathers or older brothers to baptize and confirm children in a family. AP boys usually bless sacramental bread and water in remembrance of Christ for the Sunday worship meeting.

Members are *called* to their Church roles. Newly called members, including General Authorities, are presented for raise-of-hand sustaining or opposing votes at the level associated with the calling; that is, members of a Ward would vote to sustain a woman called to serve as the Ward Relief Society President; all members of the Church would vote to sustain a man called as an Apostle or a woman called as the general Church Relief Society President. Negative votes are investigated, not tallied. Those who vote against Church-wide callings are asked to report their concerns or objections to their Stake or District President. Members are asked to give a vote of thanks when members are *released* from callings. Men are released from MP leadership positions, but not from their Priesthood ordinations (Church of Jesus Christ, 2022g).

#### Missions

More than 1.5 million Latter-day Saints have served full-time missions. Approximately 85,000 missionaries have been in full-time service each year since 2015; 65,000 were 18- to 25-year-old, single men and women serving for 18 to 24 months; 15,000 to 20,000 were retirees, usually married couples, serving for 6 to 24 months. All serve at their own expense where called to serve by the Quorum of Twelve Apostles. There are currently 411 missions in 145 countries, each supervised by a lay Mission President (Church of Jesus Christ, 2022a). The Church’s proselyting manual, *Preach My Gospel,* is available at www.churchofjesuschrist.org (Church of Jesus Christ, 2022k).

Latter-day Saint clients might offer a non-Latter-day-Saint therapist a copy of the *BofM* or suggest that the therapist meet with missionaries. It is unlikely that declining such an offer will offend a Latter-day Saint client or interfere with therapy, as Latter-day Saints have many of their missionary invitations declined.

The Church has paid employees in a wide range of professional and occupational positions. Instructors and staff in the Church’s education system are probably the largest group of Church employees (Flake, 2020). Members sometimes donate time in occupational roles, often as part-time or full-time missionaries

#### Donations

Members are instructed to “pay one-tenth of all their interest annually” to the Church (*D&C*, 1838/2013, 119:4). Tithing donations are used for buildings, salaries, congregation budgets, the Church education system, and humanitarian relief. Members are encouraged once a month to fast for 24 h if their health allows and then donate the value of the meals for those in need. Members also donate time and money for humanitarian activities.

#### Meetings

Members meet for two, one-hour meetings on most Sundays. The Sunday focus is a Sacrament Meeting, devoted to a sacrament of bread and water taken in remembrance of Christ. After this ordinance or rite, usually conducted by AP boys, members are invited to give religious talks or perform religious music. During the other Sunday hour members attend Priesthood, Relief Society, Young Women, Primary, or Sunday School meetings. There are often weeknight activities for teenagers. The Church also holds regional and Church level conferences. Congregational meetings, classes, and conferences are open to all (Church of Jesus Christ, 2022g).

#### Temples

Exaltation requires certain ordinances and rituals. For example, because Christ was baptized by John the Baptist, baptism performed with John the Baptist’s same priesthood authority is required to join Christ in the Celestial Kingdom. Church missionaries teach of this in mortality and in the post-mortal spirit realm. Other sacred, saving ordinances are conducted only in specially built and dedicated buildings called temples. Baptism, confirmation, and ordination to the Church’s priesthood are conducted outside temples for the living. Members do genealogical research to find dead relatives, then stand in as proxies to participate in saving rituals and receive saving ordinances for the dead. Proxy ordinances are only conducted in temples for those who can be identified by name as having lived when found in reliable historical records. Vicarious ordinances are considered a service or gift the living offer those who have died; those who have died are free to decline the gift of these proxy ordinances (Church of Jesus Christ, 2022c).

Dedicated temples are reserved for sacred ordinances for living members and proxy ordinances for the dead. Temples are open for tours before dedication, but open only to members vetted by a Bishop and Stake President after dedication. The premier ordinance conducted in temples is the joining or *sealing* of wives with husbands in eternal marriage and sealing of children to parents in eternal families. Personal ordinances are performed once by living members in temples; members may return hundreds or thousands of times thereafter to act as proxies, doing ordinances for validly identified, deceased persons. Members wear ritual clothing as a reminder of temple ordinances and rituals (Church of Jesus Christ, 2022g). The Church is working to build temples wherever governments are cooperative and there are sufficient members available to perform personal and proxy ordinances.

#### Church Discipline

A council of MP leaders may meet and vote to restrict or withdraw membership because of serious sins, including abuse, adultery, apostasy, and felonies. The term excommunication is no longer used. Church discipline seeks to prompt repentance and return members to full activity and blessings, to protect victims of misbehavior, and to protect the Church’s reputation. Members may resign membership by delivering a formal request to their bishop. Members are encouraged to fellowship those who have been formally disciplined by the Church when possible and never to shun current or former members. An individual may be restricted from meetings or Church buildings because of disruptive behavior (Church of Jesus Christ, 2022g).

### Controversies

#### Donations

The Church does not disclose its finances, but declares that it follows standardized, legal accounting practices. There have been recent complaints about the Church’s accrued wealth and about how its funds are managed. The Church was recently fined $5 million by the US Securities and Exchange Commission for obscuring amounts in its investment funds (Flake, 2020; Harkins, 2021; Lovett & Levy, 2020; Noyce, 2022; Schneider, 2022). A Church spokesman responded by stating that the Church had relied on trusted legal advice for the complex task of managing its money, but admitted the errors, and paid the fine (Metz, 2023).

General Authority salaries come from for-profit, taxed investments, not from donations. There is no evidence that General Authorities have lavish lifestyles. Unauthorized, but credible leaks from 2017 disclosed annual salaries of $127,000 for all General Authorities, including the governing First Presidency. This was a relatively modest salary for the chief operating officers of a large, international organization, even a non-profit organization. General Authorities have work-related expense accounts which have not been leaked (Stack, 2017).

#### Gender Inequality

While there is gender equality in deity (Mother in Heaven), there is controversy about gender status in the earthly Church. All men, but only men, may be ordained to the Church’s two priesthoods. It was assumed that women in the early Church would manage farms and businesses as their husbands served full-time missions. Other women in the early Church were called to leave Utah, study, and return with skills such as medicine (Beeton, 1978). Utah was second to Wyoming by just three months among all US states and territories in granting women’s suffrage in 1870. In 1887 federal anti-polygamy laws disenfranchised Latter-day Saint women in polygamous marriages (Arrington & Bitton, 1992).

Sonia Johnson, a Latter-day Saint member of the American Psychological Association (APA), denounced Church leaders for opposing the Equal Rights Amendment (ERA) to the US Constitution. At the 1979 APA convention she called for a boycott of missionaries (Johnson, 1979). She was excommunicated with a Church spokesman explaining that members were free to support the ERA, but not to attack and undermine the Church (Church of Jesus Christ, 1980). In 2013 Kate Kelly, a former Church missionary, formed the group Ordain Women; she was later excommunicated for highly publicized protests at Church headquarters (Stack, 2016).

From 1967 to 1978 women did not pray in Sacrament Meetings. Women now pray and speak in all meetings, participate in Ward Councils, serve as witnesses for rituals (Weaver, 2019), and serve on the Church Education System Board of Trustees (Church of Jesus Christ, 2022g). Approximately 40% of full-time missionaries are women. Gordon B. Hinckley, the 15th Prophet, encouraged women to complete all the education they could (Funk, 1996).

#### Racism

Racism has been and is present in the Church, but is officially decried as sinful (*BofM*, 1830/2013, 2 Nephi 26:33; Church of Jesus Christ, 2022g). Black men were ordained and black individuals participated in Temple rituals in the early Church. Joseph Smith advocated emancipation through government purchase of enslaved persons. Brigham Young established a policy restricting Black persons from the Church’s priesthoods and temple rituals after Utah was created as a slave-holding US territory (Stack & Noyce, 2023). Spencer W. Kimball, the 12th Prophet, ended this policy with a 1978 proclamation that is canonized (*D&C,* 1978/2013, Proclamation 2). Church membership and its leadership are increasingly diverse and the Church and the National Association for the Advancement of Colored People are officially allied to combat racism and discrimination (McCombs, 2021).

#### Vicarious Temple Ordinances

The Church compiles, archives, and organizes historical records to help identify people who have died for whom proxy temple ordinances could be performed. Proxy ordinances are conducted with little oversight or control beyond asking members to attest that historical records have correctly identified individuals for whom ordinances are to be conducted. Jewish groups objected when it was discovered that proxy ordinances had been conducted for victims of the Holocaust identified from Nazi records (Thiessen, 2005). The Church responded by cancelling ordinances for persons identified from these records for whom a family connection to the dead person could not be confirmed; by deleting more than 300,000 records entered directly from Nazi archives to its family history record system; and by established a policy limiting proxy ordinances to dead persons whose names were found and made available for ordinances by descendants. Jewish groups have complained that proxy ordinances conducted by non-family descendants have continued (Stack, 2010).

Proxy ordinances are recorded in a centralized record system, but because research, name submission, and proxy ordinance work are individual efforts, the relatives-only policy depends almost entirely on voluntary compliance by individual members. In any case, members could assert their right to conduct proxy ordinances for anyone identified and validated through genealogical research to be a dead relative, regardless of others’ objections, including objections from other family members (Church of Jesus Christ, 2022c).

## Religious and Spiritual Techniques (RSTs) for Latter-Day Saints

### Cultural Sensitivity and Competence

The APA and other mental health organizations declare multicultural awareness and sensitivity to be fundamental components of professional competence (APA, 2017a; 2017b). We believe REBT’s focus on absolutistic iBs offers an advantage in working with religiously diverse clients. Most religious problems can be treated as Activating events, conferring a degree of therapeutic neutrality toward religious issues. For example, most sins can be treated as Activating events, allowing a focus on iBs about sin, rather than on sin itself. The REBT goal would be to D, Dispute iBs *about* sin, not to D, Dispute whether a particular act is or is not sinful.

For example, the Word of Wisdom prohibits coffee, one of the most popular drinks in the world. The Word of Wisdom and its prohibition on drinking coffee can be treated as the Activating event, allowing an REBTer to focus on and dispute iBs about the Word of Wisdom. For example, if a client believes, “*I must not like the taste of coffee!*” the focus would be on replacing the *must* with a dislike that the taste of coffee is tempting rather than a demand that one not be tempted or temptable. If a client believes in Christ, it could be quite helpful to note that after his long fast, Satan tempted Christ to turn stones to bread. Bread could tempt Christ only if he wanted to eat. If a client believes something like, “*It’s terrible that I drank coffee!*” the focus would be on replacing *terrible-izing* with something like, “*It’s sad that I drank coffee.*” If a client believes, “*Coffee should not be forbidden!*” the focus would be on accepting that some rules cannot be changed rather than on demanding that God or the Church change the Word of Wisdom. If a client believed “*Drinking coffee makes me a worthless sinner!*” the goal would be to help a client remember scriptures that show that Jesus Christ told those who sinned to stop sinning without condemning or devaluing them.

REBT is preferentially persuasive and emotionally evocative (Ellis, 1994). Nielsen et al. (2001) proposed that a religious client’s dedication to scripture and theology can catalyze disputation by pre-loading RSTs with evocative persuasiveness. We find that fundamental elements of Latter-day Saint theology and scripture often explicitly dispute demanding, awfulizing, frustration intolerance, and human rating.

Latter-day Saints have the aspirational goal of being their own theologians, so that everyone “might speak in the name of God the Lord, even the Savior of the world” (*D&C*, 1831/2012, 1:20). The first lesson in the Church’s 2023 Sunday School manual emphasizes this goal, “We are responsible for our own learning” (Church of Jesus Christ, 2022e). Our Latter-day Saint clients seem not to find it unusual that we offer to discuss scripture or doctrine during therapy. A non-member therapist quoting or discussing Latter-day Saint scripture or doctrine might surprise a Latter-day Saint client, but it would not likely be objectionable, so long as the client agrees to discuss religion. A statement such as, “Do I have it right that in the *Book of Mormon* it says something like ‘Men are that they might have joy?” would probably be both disarming, somewhat intriguing, or even inviting to a Latter-day Saint client.

Most clients—more than 95%—at our clinic declare themselves to be observant Latter-day Saints on their intake questionnaires. Questions such as, *Are you a believer?* and *Could we read something in the scriptures about this?* are usually sufficient preparation to begin using RSTs with Latter-day Saint clients.

### Examples of RSTs with Latter-day Saints

Until explicated in the ABC model, iBs are usually subconsciously automatic (Ellis, 1994). Rational ideas in scripture and theology similarly hide in plain sight until clarified during REBT. These excerpts come from sessions with Latter-day Saint clients who agreed to discuss scripture. Clients are de-identified.

#### Demanding and Frustration Intolerance

Jonah (pseudonym) had successfully gathered and analyzed data for his doctoral dissertation. He had procrastinated writing and was now into an extra semester with the extra tuition costs arising because of his procrastination. He was avoiding writing. Discussing agency in the Plan of Happiness helped re-motivate him:

Nielsen (N): You wrote in the intake questionnaire that you are having trouble finishing your dissertation. What are you telling yourself about your dissertation?

Jonah (J): I really need to get to work. I need to stop procrastinating and wasting time.

N: Interesting. I read that you served a mission? Do you consider yourself a believer?

J: Yes, in Mexico, and yes, I’m an active member [of the Church].

N: Would it be okay to discuss your beliefs here?

J: Sure.

N: You spoke Spanish on your mission? How did you say this in Spanish? “You *need to,* you *have to,* you *should,* you *must* go to the Celestial Kingdom.”

J: I never said anything like that.

N: Really? How *would* you have said it if you *had* said it?

J: Something like, *Debes ir al Reina Celestial*. But I never said anything like that. It’s not true. It’s a choice, it’s not something people have to do.

N: Then why tell yourself you *need* to work on your dissertation? If you don’t have to go to the Celestial Kingdom, you sure as *hell* don’t have to work on your dissertation!

J: Okay [laughing]... Okay, but how does that help me with my dissertation?

N: Why try so hard to get into the Celestial Kingdom if you don’t have to? Think of how *damn expensive* tithing is. [He laughed again.] Why donate 10% if you don’t *have to*?

J: Because I want to go to the Celestial Kingdom.

N: Really? *Want to?* Fascinating! Whose plan was it that you would *have to* go to heaven?

J: Okay... Satan’s.

N: *Need to, have to, should, must* versus *want to.* Can you feel a difference?

J: Sure. “*Want to*” feels... lighter.

N: How’s this feel? “Jonah! You *must* work on your dissertation! You *have to* do it!”.

J: Kind of annoying.... Sorry.

N: No. It’s great that it annoyed you. “*I have to write*,” versus “*I want to write?*”.

J: “*I want to*” is more motivating.

N: *Do you* want to work on your dissertation?

J: Yes and no. Sometimes I do, sometimes I don’t.

N: Welcome to the human race! But what do you *really* want?

J: I want to get it done as soon as I can.

N: *Really? Good!* How about this? Listen for *shoulds* so you can stop *shoulding* on yourself. [J smiled.] Oh, you got that little joke. Listen carefully for when you and others say they *should*, *have to*, *got to*, *need to*, or *must* do stuff. Listen for when people *must... turbate*.

J: What? Oh [laughing]!

N: *Every time* you hear yourself or someone else say, *have to*, *need to*, *got to*, *ought to*, *should*, *must*, or other demanding things, say something like this *to yourself*: *Hey stop shoulding on yourself!* or, *Listen to that: musturbating in public*! Remind yourself that you *want to* but *don’t have to* work on your dissertation. Why? You *want to* but *don’t have to* go to the Celestial Kingdom, so you *don’t have to* finish your dissertation! *You want to!*

Jonah committed to insist to himself that he did *not* have to, but *did want to* work on his dissertation and that he no longer *wanted* to delay. He deleted social media and streaming apps from his computer and smart phone and committed to three, daily, 15-min bouts of writing. He almost always spent more than 15 min writing once he started writing. He completed his first full draft in three weeks, worked out revisions with his chair and committee, then successfully defended the dissertation before the end of the semester.

Perhaps 2% to 5% of Latter-day Saint clients are not amused by mild vulgarities or REBT’s neologistic jokes such as *shoulding on one’s self* or *musturbation*. Easing into humorous disputation usually prevents problems with such humor. Jonah seemed quite relaxed from the beginning of the session on and the guess that a humorous approach would further relax him and help him accept REBT proved to be correct.

#### Awfulizing and Frustration Intolerance

Martha (pseudonym) wrote in her intake questionnaires that she attended two semesters at our university before her mission, earning a 3.96 cumulative grade point average (GPA); this was at the 96^th^ percentile for the university. She returned from her mission, restarted classes, performed poorly on several mid-term tests, grew discouraged, and stopped attending classes. She failed all her classes that first semester back from her mission. Her GPA dropped from 3.96 to 2.64; she lost a scholarship and was placed on academic warning by the university. She came for help early in the next semester. Martha wrote on one of the intake questionnaires that she now could “never get into graduate school.” She slept in on the day of the intake, arriving 15 min late for the appointment.

N: I read what you wrote in the intake questionnaires about graduate school. Could we begin by you telling me what you’re telling yourself about grad school?

Martha (M): My GPA will never recover. I’ve *wasted my time!*

N: Wasted your time.... Let’s use the ABC model right here: A, your Adversities are these: First, last semester’s grades lowered your GPA; second, you lost your scholarship; and third, grad school seems impossible. Do I have that right? [M nodded.] Now you B, Believe, you’ve wasted your time. The C, emotional Consequence, of B is that you feel so discouraged you often can’t get out of bed.

M: Yes, it all feels like a waste of time.

N: Well, it *feels* like a waste of time because you *B*, *Believe* it’s a waste of time. Let’s ABC*D*, *Deliberate* about that waste-of-time belief. You just returned from a mission. Are you a believer?

M: Yes! That makes it *worse!*

N: *Worse?* How?

M: I’ve been wasting the Lord’s time, *too!*

N: Wasting the Lord’s time, too.... Can we discuss what the Lord thinks about wasting time?

M: Okay...

N: Do you remember what the Lord told Joseph Smith about his four months in Liberty Jail?

M: I’m not sure. We did a [family] Church-history, road trip before my mission. First Liberty Jail [in Missouri], then Carthage Jail [in Illinois, where Joseph Smith was murdered] and Navoo [in Illinois], then the Kirtland Temple [in Ohio], the Sacred Grove [in New York, where Joseph Smith had his first vision] and the Hill Cumorah [in New York, where Joseph Smith received the golden plates]. Four months in Liberty Jail?

N: A bit more than four months. Interesting that his time in Liberty Jail and a semester here are about the same length. [M smiled.] Would you read what the Lord told him?

M: [From Nielsen’s *D&C*] “Know thou my son, that all these things shall give thee experience, and shall be for thy good” (*D&C*, 1839/2013, 122:7).

N: Were Joseph Smith’s four months in Liberty Jail a waste of time?

M: I guess not.

N: What if you believed that last semester’s four months were *not* a waste of time?

M: I don’t know. I wish I could believe it. I suppose I’d feel better.

N: Do you remember what Alma the Younger said about comparing a belief to a seed?

M: Something like, treat a belief like a seed; try to believe; if it grows you’ll see if it’s a good belief.

N: Right. Would you read what Alma said? Alma [chapter] 32: [verse] 27 [in the *BofM*]?

M: [From Nielsen’s *BofM*] “If ye can no more than desire to believe, let this desire work in you, even until ye believe in a manner that ye can give place for a portion of my words.”

N: Of course, Alma the Younger was a prophet. I’m a wicked old shrink [M chuckled]. But could you plant the seed of believing that your adversity is *not* a waste of time and *might* benefit you?

M: How?

N: Well, first, water and feed the seed by *forcefully* telling yourself something like, “I really, *really* hope that I can get into grad school, but even if I *don’t*, my life is *not* a waste of time*.* It would be rotten, but it *could* give me valuable experience. *Not* experience I want, but it *could* be for my benefit.”

M: Okay.

N: Add something like, “My best chance is to work... like... *heaven* [M smiled] to get better grades and bring my GPA up.” And say it *as if you believe it,* even if you *don’t*. Try it... now... out loud.

M: Okay... I *really* want to get a Ph.D. in clinical psychology, but my life is *not* a waste if I don’t! And my *best chance* is to *work hard!*

N: Good work! Now *shout it*... [her eyes got wide]... in your head. [She closed her eyes briefly.] What was that like?

M: It felt more believable. I could try it.

Martha attended 10 more sessions that semester, during which we continued to discuss her beliefs, emotions, motivations, and study strategies. She earned all “A” grades at the end of that semester, raising her cumulative GPA to 2.96, a B- average. She is still considering applying to a doctoral psychology program and plans to return from time to time to discuss her motivation, her goals for graduate school, and her progress.

#### Human Rating

Damaris (pseudonym) was infatuated with Luke (pseudonym) a young man in her Latter-day Saint ward. She was afraid to flirt with Luke.

N: What are you telling yourself about flirting with Luke?

Damaris (D): What if he’s not interested?

N: What if he’s *not?* What are you telling yourself about Luke not being interested in you?

D: Maybe I’m not worth his time.

N: Now we can see why you won’t flirt: A, the Activating event is that you think he may not be interested. Then you B, Believe, that if he’s not interested, *you’re* not *interesting*. The C, emotional Consequence of B, your Belief, is anxiety shuts you down. So Luke determines human worth?

D: Well... I know that’s not right, but that’s how it feels.

N: It feels like that because you *believe* it. You *strongly believe* that your worth comes from what Luke and perhaps what other people, perhaps especially men, think of you. What could you believe, instead?

D: That Luke doesn’t determine my worth?

N: Right. You know that’s what I want you to say. Could you say it with *less* conviction?

D: [Laughing.] I’m not sure. Probably not.

N: You said you and Luke are in the same Ward. Are you a believer?

D: Yes...

N: Would you like to see what the Lord says about Luke?

D: Okay. About Luke?

N: Well, not Luke by name, but it’s kind of about Luke and about what you believe about Luke’s opinions. What does the Lord say about your worth? You may even have this memorized.

D: “Remember, the worth of souls is great in the sight of God?” [*D&C*, 1829/2013, 18:10].

N: Right! Word for word, I think. If Luke doesn’t want to kiss your toes, does he overrule God?

D: [Laughing.] No.

N: Good, but you’re not convinced of that. Do you have the *next* verse memorized? Verse 11? [M shook her head.] I do—both 10 *and* 11. Check me: “Remember, the worth of souls is great in the sight of God;” that’s verse 10, notice, it ends with a semicolon. What does the semicolon mean?

D: That the sentence and thought continue.

N: Hey, you must be a college student! [D smiled.] Check me on the whole sentence: “Remember, the worth of souls is great in the sight of God;” *semi-colon, “*For, behold, the Lord your Redeemer suffered death in the flesh; wherefore he suffered the pain of all men, that all men *might repent* and come unto him*”* [*D&C,* 1829/2013, 18: 10 & 11, extra emphasis spoken]. “*Might repent*.” Any evidence there that worth goes down if a person *willfully refuses* to repent? Willfully *sins* and then *refuses* to repent?

D: No, sin doesn’t change the worth of a soul.

N: Luke’s opinion must be really, *really, really, very, very* important if his interest or disinterest in you changes your worth! It says, “In the sight of God.” So, God’s point of view or Luke’s point of view?

D: God’s point of view, of course.

N: What could you tell you about your worth and Luke’s opinion? Say it with force this time.

D: Luke does *not* determine my worth!

N: Hey, pretty good! Does that feel more or less like you could flirt with Luke?

D: More. But actually, I felt a little annoyed when I said it. I don’t know why.

N: Yikes! Of course, we don’t yet know what Luke thinks about romance with you. Maybe he hasn’t noticed you. Maybe he’s *anti*-noticing you—trying not to notice you. How about if you add something like this? “If Luke is *not* interested, *his loss.”* Of course, it would be your loss, too*,* but *you don’t lose more than Luke loses.*”

Damaris agreed to talk to herself in this way before the next Sunday, when she would see Luke again. She role-played flirting with Luke and did flirt with him. He seemed indifferent. The next week she agreed to invite him to come to Sunday dinner at her apartment. He politely declined and she felt mildly annoyed. She accepted Nielsen’s challenge to flirt with other men and began actively inviting men to Sunday dinner. Five weeks later she began dating Mark, one of three men, John, Mark, and Matthew (pseudonyms) she had to invited to Sunday dinners. She had invited Mark and Matthew on the *same* Sunday to balance out the number of men and women at that dinner.

### Effectiveness

We selected excerpts that seemed to be successful examples of using Latter-day Saint scripture and theology as RSTs during REBT. “Cherry-picked” sessions might make any therapy look good, so we compared symptom change among our clients with symptom change among clients treated by colleagues with no allegiance to REBT. We cannot determine exactly which of our clients got RSTs, but RSTs were our preferred modus operandi with observant Church members and more than 90% of our clients declared themselves observant.

#### Symptom Change

Our clinic uses the 45-item Outcome Questionnaire (OQ; Lambert et al., 2004) to assess symptoms. We, the four authors, of this paper, compared improvement scores, computed as OQ_1_ minus OQ_last_, among 4,732 clients we treated, with the improvement scores of 41,027 clients treated by 402 non-REBT colleagues (69 professionals, 333 trainees). Our clients improved more: *M*_ChangeREBT_ = 8.51 (*SD* = 22.15) versus *M*_ChangeOthers_ = 5.16 (*SD* = 20.21), *t* (45,757) = 10.11, *p* < .001.

**Fig. 2:**
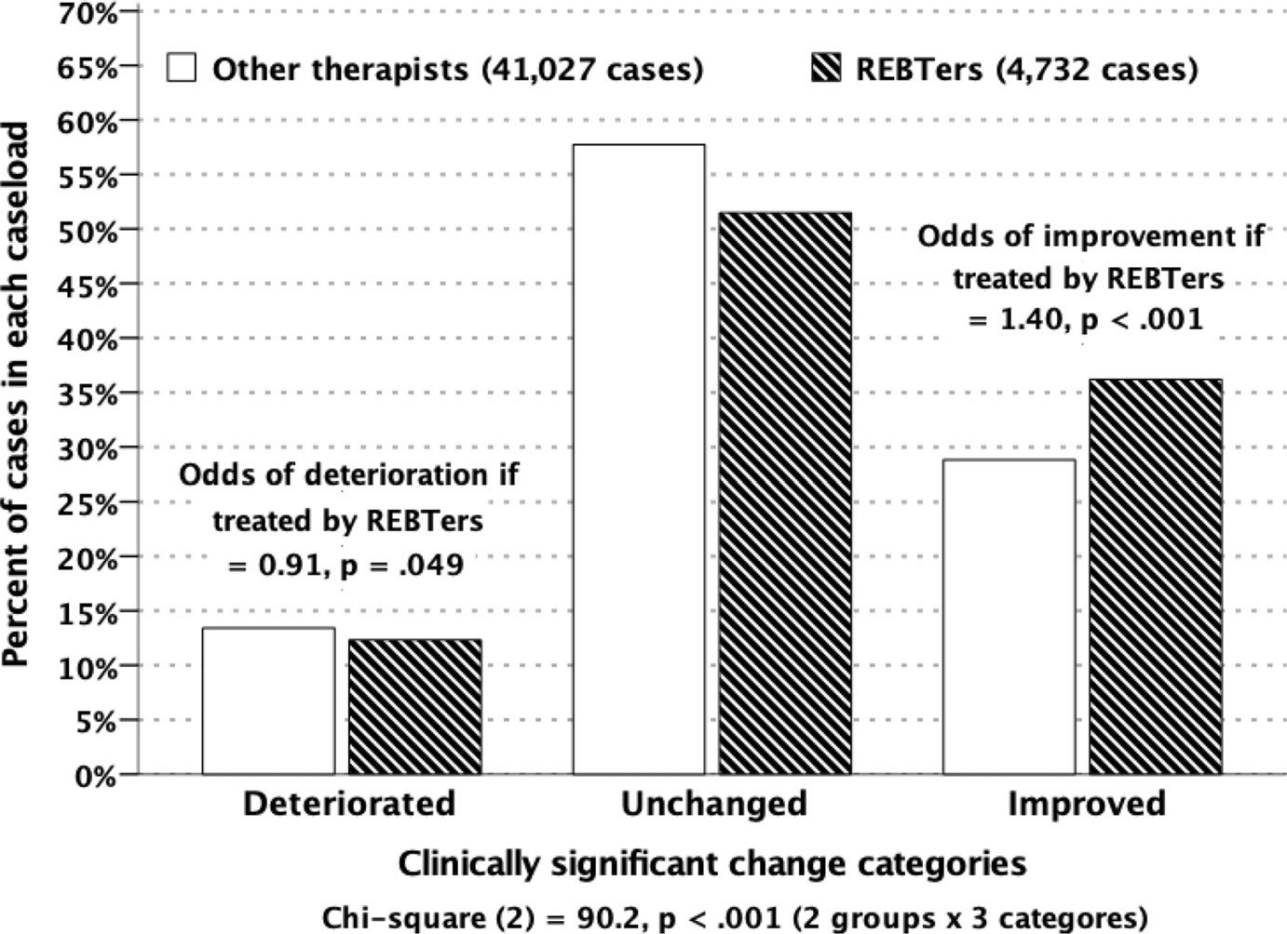
Percent of Clients Classified Significantly Improved, Significantly Deteriorated, or Unchanged, in Caseloads of REBTers and Other Therapists

Pairs of OQ scores are expected to change by 14 or more points for fewer than 5% of change scores (Lambert et al., 2004). This yields three change categories: significantly improved, unchanged,  and significantly deteriorated. Figure 2 shows percentages of clients in these three categories for our clients and for clients treated by other therapists. The distributions are significantly different: χ^2^ (2) = 90.20, *p* < .001. Our clients were 40% more likely to improve and 9% less likely to deteriorate: The improvement *odds-ratio* for REBTers was 1.40, χ^2^ (1) = 98.91 (*p* < .001). The deterioration *odds-ratio* for REBTers was 0.91, χ^2^ (1) = 3.88 (*p* = .049).

#### Scriptures and Improvement

Funding from the Templeton Foundation allowed us to more directly study the effects of using Latter-day Saint scripture as an RST (Nielsen, et al., 2023). A web-based application developed with Templeton support was used to code session-by-session therapy events; 61 therapists at our clinic coded 5,865 elements of sessions they provided to 759 clients, including 542 instances of discussing Latter-day Saint scripture. After controlling for pre-treatment OQ score, each use of a scripture predicted a 1.11-point reduction in adjusted OQ score at follow-up: OQ_follow-up_ = 33.0 + (0.50 * OQ_pretreatment_) − (1.11 * *N* of scriptures); *F* (2, 747) = 129.4, *p* < .001; *t* (747) for *B*_scriptures_ = 2.23, *p* = .023, Cohen’s *d* = 0.25. The *d* value indicates that use of scripture yielded one-quarter of one standard deviation’s worth of extra improvement; put another way, if 30 of 100 clients would generally improve, 9 additional clients would improve when scriptures were used as an RST.

Therapists could code theoretical orientation in each session. Orientation was not a significant predictor of change in OQ-45 scores. Other therapists besides REBTers discussed scripture, coding as their orientations for sessions acceptance and commitment therapy (ACT), cognitive behavior therapy (CBT), and compassion focused therapy (CFT). ACT, CBT, and CFT interventions overlapped with REBT interventions. Nonetheless, use of Latter-day Saint RSTs predicted improvement among our Latter-day Saint clients across orientations.

## Discussion

It seemed clear to us that many Latter-day Saint scriptures and doctrines dispute iBs associated with demanding, awfulizing, frustration intolerance, and human rating. Furthermore, fundamental elements of Latter-day Saint theology support healthy rBs: Countering demands, God gives humans moral agency. Countering awfulizing, opposition in life is part of mortal development. Countering frustration intolerance, mortal development requires effort. Countering human rating, sin does not lower human worth. Our clinical experience suggested that Latter-day Saint scripture and doctrine can be used to construct REBT disputations—RSTs—for use in REBT conducted with observant Latter-day Saint clients. Symptom change patterns at our clinic supported our impressions.

### Religious Coping

RSTs in REBT might be seen as overlapping with and augmenting positive religious coping or as counteracting negative religious coping (Pargament, 1997). Pargament defined religious coping as positive and negative manifestations of five broad religious functions: (1) finding meaning; (2) finding a sense of control; (3) finding comfort; (4) finding a perception of cohesiveness; and (5) facilitating and understanding transitions. Pargament and colleagues then developed an instrument to measure negative as well as positive manifestations of these functions, the RCOPE (Pargament et al., 2000). Jonah, Martha, and Damaris, from whose therapies we drew excerpts, did not complete the RCOPE, so we cannot clearly identify elements of religious coping in their situations or responses to REBT. We can speculate.

Jonah’s avoidance of writing revealed the paradoxical impact of demands. He was *de*motivated by the idea that he *should* write. Nielsen’s quick intervention precluded our seeing his negative religious coping. He quickly agreed that demands are satanic and moral agency is holy, moving him toward Control as a means of religious coping, evident in the RCOPE’s Self-Directed Religious Coping subscale (SDRC; Pargament et al., 2000, p. 523). His new approach overlapped somewhat with SDRC subscale items. The SDRC item closest to Jonah’s shift is likely, *Tried to deal with the situation on my own without God’s help* (p. 523). Jonah’s motivation improved when he viewed his dissertation as a choice.

Remember that Martha chastised herself for wasting the Lord’s time. This thinking is a negative religious coping pattern that seems to fit what Pargament et al. (2000) called a Punishing God Reappraisal among the Finding Meaning RCOPE items (p. 522). When she read what Christ told Joseph Smith in Liberty Jail, “Know thou my son, that all these things shall give thee experience, and shall be for thy good” (*D&C*, 1839/2013, 122:7), the REBT goal could be described as attempting to move her thinking toward Benevolent Religious Reappraisal. Two items from this RCOPE subscale seem a good fit with this RST: *Tried to find a lesson from God in the event. Tried to see how God might be trying to strengthen me in this situation* (Pargament et al., 2000, p. 522).

Damaris’s difficulties came from rating herself based on how Luke and other men *might* view her. Self-rating is so common among our clients that it is surprising when self-rating is not tied to their self-defeating emotions. Reminding Damaris of God’s unconditional acceptance of all humans, including people who willfully refuse to repent, moved her toward unconditional self-acceptance (USA). USA helped her flirt and ask Luke out. She continued to accept herself when he was uninterested, when she asked other men out, and when, in short order, she began dating Mark. Negative coping items on the Spiritual Disconnect subscale for the RCOPE may have described her views before she developed USA: *Questioned God’s love for me. Wondered if God really cares* (Pargament et al., 2000, p. 523).

### Future Directions for RSTs in REBT and CBT

We hope the larger community of REBTers and CBTers will report on their work with clients from different faith traditions, and especially hope for descriptions of their religious clients’ varied beliefs and the RSTs they use with these clients. Describing the distinctive histories, theologies, scriptures, and wisdom literatures of different faith traditions, with a focus on rBs derived from scripture and wisdom literature, will increase RSTs available to REBTers and CBTers, and will enlarge our therapeutic armamentarium.

A more ambitious project would be to develop a multisite project like the Bridges Consortium for the study of Spiritually Integrative Psychotherapy funded by the Templeton Foundation (Nielsen et al., 2023). One goal would be to link specific interventions with outcomes. We were able to detect a reliable relationship between Latter-day Saint scripture and symptom reduction using the web-based application developed by the lead Templeton Foundation researchers. The app allowed simple, quick (three to five minutes), session-by-session coding of interventions, including RSTs. Researchers in a separate arm of this Templeton funded project used the app to measure session-by-session symptom scores and code Gestalt Pastoral Care (GPC) RSTs. Their group reported the first empirical findings supporting the effectiveness of GPC (Thomas et al., 2022).

In our arm of the Templeton-funded research we found that REBT lends itself to straight-forward coding of discrete steps in REBT interventions like the steps described by Dryden et al. (2010). It was relatively easy to code kinds of disputations (e.g., rational alternative disputations, logical disputations, etc., didactic style, Socratic style, etc.), and easy to code specific RSTs (e.g., to code D&C 18:10 & 11 supporting the rB that God does not devalue sinners, as used above, or to code the disputation used with Jonah, above, that *Satan uses shoulds*). The global REBT community might adopt this web-based application or a similar web-based app to measure session-by-session symptom change and code session-by-session REBT interventions. This could yield a large, multinational, practice-based archive through which we could link outcomes with specific REBT interventions, including specific RSTs.

## Footnotes

1 To avoid confusion about the role of Jesus Christ in its theology, the Church has asserted a preference not to be called the Mormon Church, Latter-day Saint Church, or LDS Church. The preferred editorial style is to first write, *Church of Jesus Christ of Latter-day Saints*, and thereafter, when the context is clear, *the Church*. Also, *members of the Church*, *Latter-day Saints*, or simply *members* are preferred over Mormons (Church of Jesus Christ, 2022n). Latter-day Saints will not take offense at being called Mormons.

2 For brevity, we shall hereafter sparingly write, *Latter-day Saints believe* and *members believe*. Assume we intend to describe the Church and its doctrines, not act as apologists.

3 Some members remained behind in the Midwest and formed offshoot churches when the Quorum of Twelve Apostles moved the Church to the Great Basin. The largest of these offshoots, with approximately 250,000 members in 51 countries, is the Community of Christ, headquartered in Independence, Missouri (Community of Christ, 2018). The Community of Christ claims authority through Joseph Smith’s descendants. Other offshoots are much smaller, with no more than 70,000 current members altogether.

4 At least 10 later offshoot churches claimed authority to practice polygamy after Wilford Woodruff’s 1890 proclamation ending the practice in the Church. Despite sensational publicity, these offshoot polygamous churches have no more than 20,000 members, altogether.
